# Anxiety among healthcare workers during the COVID-19 pandemic: a longitudinal study

**DOI:** 10.3389/fpubh.2023.1236931

**Published:** 2023-11-30

**Authors:** Esmee Bosma, Verena Feenstra, Sandra H. van Oostrom, Lifelines Corona Research Initiative, H. Marike Boezen, Karin I. Proper

**Affiliations:** ^1^Center for Prevention, Lifestyle and Health, Department Behavior and Health, National Institute for Public Health and the Environment, Bilthoven, Netherlands; ^2^Department of Public and Occupational Health, Amsterdam UMC, Vrije Universiteit Amsterdam, Amsterdam Public Health Research Institute, Amsterdam, Netherlands

**Keywords:** healthcare workers, anxiety, mental health, COVID-19 pandemic, longitudinal data

## Abstract

**Background:**

During the COVID-19 pandemic, many healthcare workers faced extreme working conditions and were at higher risk of infection with the coronavirus. These circumstances may have led to mental health problems, such as anxiety, among healthcare workers. Most studies that examined anxiety among healthcare workers during the COVID-19 pandemic were cross-sectional and focused on the first months of the pandemic only. Therefore, this study aimed to investigate the longitudinal association between working in healthcare and anxiety during a long-term period (i.e., 18 months) of the COVID-19 pandemic.

**Methods:**

Data were used from online questionnaires of the Lifelines COVID-19 prospective cohort with 22 included time-points (March 2020–November 2021). In total, 2,750 healthcare workers and 9,335 non-healthcare workers were included. Anxiety was assessed with questions from the Mini-International Neuropsychiatric Interview, and an anxiety sum score (0–7) was calculated. Negative binomial generalized estimating equations (GEE), adjusted for demographic, work and health covariates, were used to examine the association between working in healthcare and anxiety.

**Results:**

Anxiety sum scores over time during the COVID-19 pandemic were similar for healthcare workers and non-healthcare workers. No differences between the anxiety sum scores of healthcare workers and non-healthcare workers were found [incidence rate ratio (IRR) = 0.97, 95% CI = 0.91–1.04].

**Conclusion:**

This study did not find differences between healthcare workers and non-healthcare in perceived anxiety during the COVID-19 pandemic.

## Introduction

Since the outbreak of the COVID-19 pandemic in March 2020, healthcare systems in many countries have been struggling to offer adequate care to all patients (1, 2). The large number of COVID-19 cases and the risk of death of those who were infected led to a high demand for medical care. This increased demand for care also meant that care capacity and resources reached their limits. Many healthcare workers were faced with a high workload, high work pace and long shifts (1, 2). In addition, healthcare workers were at an increased risk of being infected with SARS-CoV-2 when caring for COVID-19 patients (1, 3). The extreme working conditions and the high infection risk during the pandemic may have led to emotional distress and may have negatively affected the mental health of healthcare workers (1, 2).

During earlier virus outbreaks, healthcare workers faced several risk factors for mental health problems, among which anxiety (4–6). During the Severe Acute Respiratory Syndrome (SARS) outbreak in 2003, risk factors for mental health problems included fear for a decline in one’s own health and the health of others, social isolation and work stress (4–6). Health concerns were caused by the fear of getting infected and infecting others. To lower the infection rate, healthcare workers had to socially isolate themselves. Higher perceived work stress was related to increased workload, changes in work tasks and tension between colleagues during the SARS outbreak (4–6).

Also during the COVID-19 pandemic, these risk factors are frequently reported as potentially harmful to the mental health of healthcare workers (7–11). In addition, concerns about personal protective equipment and feeling unprepared for the COVID-19 pandemic are identified as risk factors for mental health problems in healthcare workers (7–9, 11). Because of these severe psychosocial working conditions for healthcare workers, it is plausible to expect a higher prevalence of mental health problems, amongst others anxiety among healthcare workers compared to workers in other sectors. However, research to this is currently limited. The present study compares healthcare workers with workers in other sectors and focuses specifically on anxiety as an important mental health condition, because for many healthcare workers it was not possible to keep their distance from the patient, which could in turn lead to experiencing anxiety of becoming infected themselves or contamination for vulnerable patients or family members. The lack of personal protective equipment among some healthcare workers might additionally have increased anxiety.

Several systematic reviews revealed that a large proportion of healthcare workers suffered from anxiety during the COVID-19 pandemic (7, 9, 10, 12, 13). The majority of studies on which these reviews are based include an Asian, mostly Chinese, population. A systematic review of Li et al. (12), which was published during the pandemic and includes studies from various world regions, reports a pooled prevalence of 7.9% (95% CI = 4.4%–12.3%) for generalized anxiety disorder (GAD) among healthcare workers (based on studies with random sampling). GAD is an anxiety disorder, defined by chronic excessive worry for at least 6 months, in combination with at least three psychological or somatic symptoms (14–17). Anxiety can negatively influence work and social functioning, productivity, and quality of life among healthcare workers (18–21). Because of the association between anxiety symptoms and functioning at work, it is important to understand whether there are higher levels of anxiety among healthcare workers during the COVID-19 compared to other workers, in order for healthcare workers to be supported.

Studies that have compared anxiety levels among healthcare workers with anxiety levels among workers in other sectors, hereinafter referred to as non-healthcare workers, reveal contradicting results. A Chinese study found no difference in the occurrence of anxiety between occupational groups during the COVID-19 pandemic (3). A German study concluded that in the first month of the pandemic, the occurrence of anxiety was even lower among healthcare workers compared to non-healthcare workers (22), which was explained by the relatively high subjective levels of information regarding COVID-19 among healthcare workers (22). However, most studies that examined anxiety among healthcare workers during the COVID-19 pandemic were cross-sectional, were limited to the first months of the pandemic, used no reference group, or only used 2 or 3 timepoints (3, 7, 9, 10, 12, 22–25). Therefore, the current study aims to investigate the longitudinal association between working in healthcare and anxiety during a long-term period during the pandemic (March 2020–November 2021), where anxiety was measured in periods of high and low COVID-19 infection rates. It was hypothesized that healthcare workers experienced anxiety to a greater extent than non-healthcare workers during the COVID-19 pandemic.

## Methods

### Study design and population

Data from the Lifelines COVID-19 prospective cohort study were used. This cohort was initiated at the beginning of the COVID-19 pandemic, to examine COVID-19 infections and its health and societal impacts in the Dutch population (26). The Lifelines COVID-19 cohort is part of the larger Lifelines population cohort which is a multi-disciplinary prospective population-based cohort study examining in a unique three-generation design the health and health-related behaviors of 167,729 persons living in the North of the Netherlands (provinces Drenthe, Groningen, and Friesland) (26, 27). It employs a broad range of investigative procedures in assessing the biomedical, socio-demographic, behavioral, physical and psychological factors which contribute to the health and disease of the general population.

**Figure 1:**
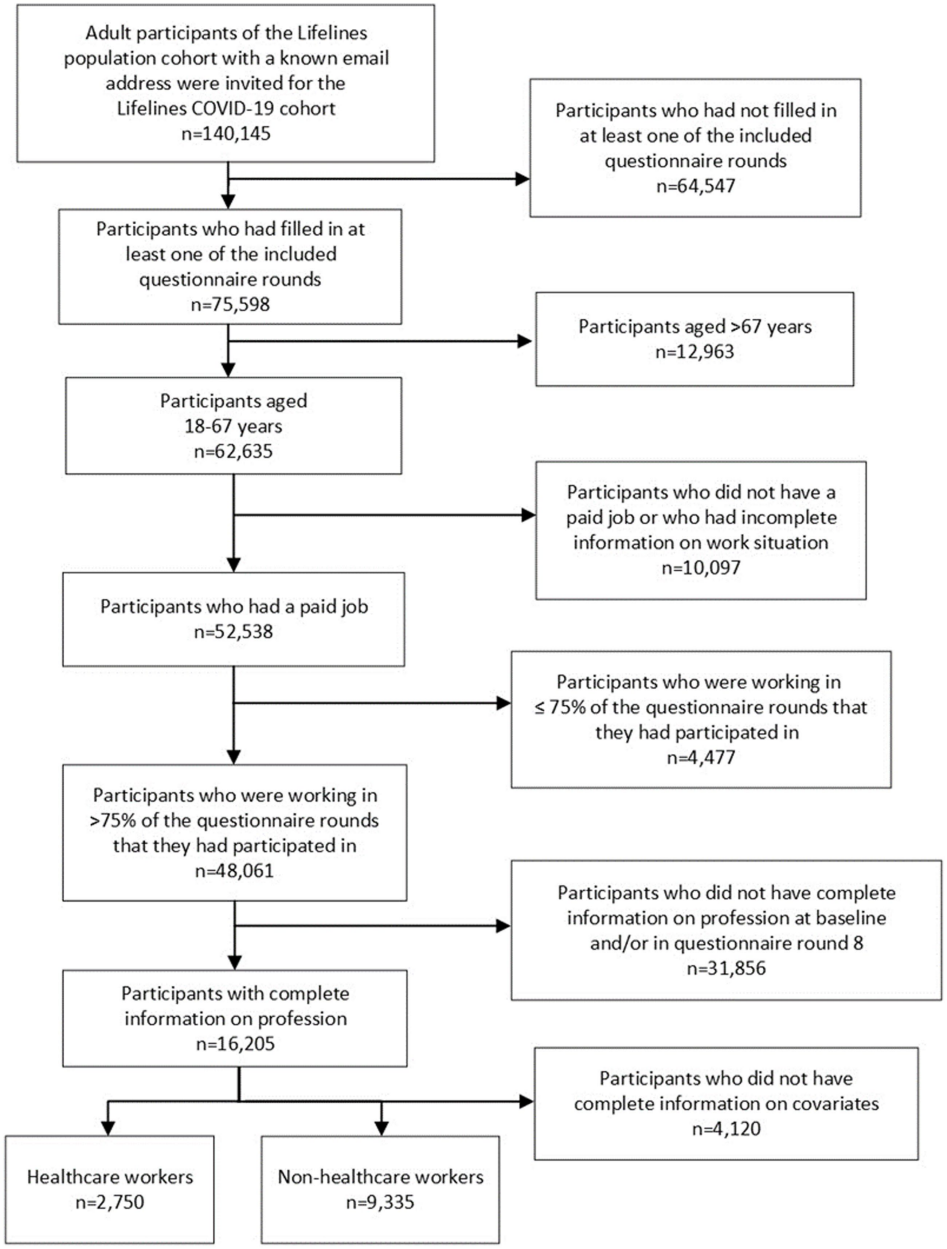
Flow diagram of the study population.

To be included in the Lifelines COVID-19 cohort, participants of the Lifelines population cohort had to be ≥18 years old, their email address had to be available (*n* = 140,145) and they had to have filled in at least one of the included questionnaire rounds (*n* = 75,598) (Figure 1) (26). As the current study focuses on workers, participants were selected if they (i) were ≤ 67 years old (*n* = 62,635), (ii) had a paid job (*n* = 52,538), (iii) worked for the majority (>75%) of questionnaire rounds that they had completed (*n* = 48,061), (iv) had complete data on their profession in the general assessments in the Lifelines population cohort and in questionnaire round 8 (in the other rounds, no questions were asked about profession) of the Lifelines COVID-19 cohort (*n* = 16,205) and (v) had complete data on all covariates (*n* = 12,085). Participants that did not meet these criteria were excluded (*n* = 128,060).

### Data collection

For data collection, digital self-administered questionnaires were used (26). These questionnaires included questions on socio-demographic characteristics, general health, chronic diseases, well-being, mental health, social relationships and lifestyle factors (26).

The first questionnaire was sent out on March 30, 2020 (26) (Table 1 in Supplementary Material). After this, new questionnaires were sent out weekly until May 18, 2020, after which the questionnaires became biweekly. From the eighth questionnaire round (May 23, 2020-June 24, 2020), participants were only invited to follow-up Lifelines COVID-19 questionnaires if they had completed at least one of the previous questionnaires. As of July 2020, the questionnaires were sent out monthly. Data of the same participants in different study rounds could be linked to each other by a pseudonymized linking variable which was provided by the Lifelines COVID-19 cohort. The current study comprises data from 22 time-points, between March 2020 and November 2021 (26). Questionnaire round 12 (24 July-2 September) was excluded, because no data on anxiety symptoms were collected in this round.

### Measures

#### Healthcare workers

To determine whether participants had a paid job, each questionnaire included a question on what participants currently do in their daily lives (I am a student; on disability; unemployed; retired; on maternity leave; work; other). Participants who answered ‘I work’ in the majority of questionnaire rounds (>75%) that they had filled in, were classified as having a paid job.

Participants were divided into healthcare workers and non-healthcare workers. Participants were considered healthcare workers, when they had indicated that they (i) had a care and welfare profession at the general assessments of the Lifelines population cohort by means of an open-ended question, professions were categorized into 13 classes (Table 2 in Supplementary Material); (ii) were working within the health services sector in questionnaire round 8 (only round 8 contained the relevant question, Table 3 in Supplementary Material) and (iii) had not started working in another occupational sector than the health services sector (‘have you changed your profession or employer in the last month?’) in questionnaire rounds 10, 13, 16, 19, 22, and/or 23 (this question was not asked in the other rounds). Participants were considered non-healthcare workers, when they had indicated that they (i) had a profession that is not related to care and welfare at the baseline measurements; (ii) were working within another occupational sector than the health services sector in questionnaire round 8; and (iii) had not started working within the health services sector in questionnaire rounds 10, 13, 16, 19, 22 and/or 23.

#### Anxiety

Anxiety was assessed with questions from the Mini-International Neuropsychiatric Interview (MINI) (26–28). The MINI is a brief structured diagnostic interview that is compatible with international diagnostic criteria as the Classification for Diseases (ICD-10), DSM-III-R, DSM-IV and DSM-V (29, 30). In the Lifelines COVID-19 cohort, anxiety symptoms belonging to a generalized anxiety disorder (GAD) were questioned with the MINI following the definition of DSM-IV (see Table 4 in Supplementary Material for all items) (31). The MINI was not conducted as a diagnostic interview, but by means of a self-reported questionnaire. The self-reported questionnaire version of the MINI has also been used in previous studies on the Lifelines COVID-19 cohort (32, 33).

The weekly and biweekly Lifelines Covid-19 questionnaires refer to the symptoms since the last observation (‘in the last 7 days’ or ‘in the last 14 days’), Consequently, the symptom duration does not match the definition of GAD (symptom duration of at least 6 months) (17). Therefore, an anxiety score was calculated for every questionnaire round based on a sum score (range 0–7) of seven anxiety symptoms: excessive worry, restlessness, tenseness, tiredness, difficulty concentrating and making decisions, irritability, and sleeping problems. Given the time between sending out the questionnaires, questionnaire rounds 1–6 measured the presence of anxiety symptoms in the last 7 days and questionnaire rounds 7–11 and 13–23 used a recall period of 14 days. All questions could be answered with either yes or no.

In rounds 1–9, one of the anxiety symptoms (tiredness) was not part of the questionnaire. Therefore, a dataset was provided in which these missing values, and limited missing values due to non-response for a specific item, were imputed single dataset imputation with Multivariate Imputation by Chained Equations package in R, as was done for the study by Ori et al. (33). Information used for imputation included age, sex, body mass index, household composition, income, profession and mental health characteristics from other time points within the Lifelines COVID-19 study and from the Lifelines general assessments. If a participant did not fill out an entire questionnaire, the symptoms were not imputed on this time point.

#### Covariates

Covariates on demographic (sex, age, education level, household composition), work (employment, working hours) and health characteristics [chronic health condition(s), chronic psychological illness, COVID-19 test result, COVID-19 vaccination] were included.

Sex was classified as female or male. Age in years at the time of the first questionnaire round was calculated using the given month and year of birth at the baseline measurements. Subsequently, age was categorized into the age groups; 18–35, 36–50, 51–67. Participants’ educational level was based on the highest level of education attained and categorized as low (no education; primary education; lower or secondary vocational education; junior general secondary education), middle (secondary vocational education or work-based learning pathway; senior general secondary education or pre-university secondary education) or high (higher vocational education; university education). Participants who are living with others could indicate how many household members of specific age groups (0–12, 13–18, 19–30, 31–60; >60) they had. Household composition was categorized into; living alone, living together with adult(s), living together with child(ren), living together with child(ren) and adult(s) and living together but unknown with whom.

Employment contract of participants was assessed in questionnaire rounds 1–10, 13, 16, 17, 19, and 21–23. The response options (permanent; temporary; zero hour, flexible, on call; freelance; other) were categorized into three groups; permanent contract, temporary contract and both permanent and temporary contract. The number of working hours per week was assessed in questionnaire rounds 8, 10, 13, 16, 19, 22, and 23. The mean of the indicated working hours at these time-points was determined.

The presence of a chronic health condition including chronic psychological illness was determined if participants indicated this in questionnaire rounds 1, 2, 14, or 22. The following chronic health conditions were measured; cardiovascular disease, high blood pressure, heart attack, narrowing of the arteries in the legs, stroke and/or tia, other heart and/or coronary disease, lung disease, liver disease, kidney disease, diabetes, chronic muscle disease, psychological illness, auto-immune illness, cancer, neurological disease, problems with spleen, other chronic condition. For each questionnaire round, participants were asked about a positive test result for a SARS-CoV-2 infection, based on testing at an organization (Municipal Health Services, work or school, access test organization, or a different organization) or self-testing. Furthermore, in questionnaire rounds 18–23, information was obtained on whether participants had been vaccinated against SARS-CoV-2. Participants were defined as vaccinated, if they had indicated in at least one of the questionnaires that they had received at least one COVID-19 vaccination.

### Data analyses

Characteristics of the study population were stratified for healthcare workers and non-healthcare workers and tested using chi-square tests and independent-sample t-tests. The anxiety sum scores over time are presented visually through a figure with the percentages of participants without any anxiety symptom (sum score = 0) and the median anxiety sum score for scores >0 for both healthcare workers and non-healthcare workers.

The longitudinal association between working in healthcare and anxiety during the COVID-19 pandemic was studied using negative binomial generalized estimating equations analysis with an exchangeable correlation structure (34, 35). The longitudinal data contain repeated observations on each subject, leading to correlation between the observations within a subject. Generalized estimating equations (GEE) account for this correlation by providing reliable estimators of the regression coefficients and the variances (34). The negative binomial analysis was chosen in order to account for the non-normal distribution of the outcome measure, which can be compared with the distribution of a count variable. The incidence rate ratios were calculated from the negative binomial regression coefficients by exponentiating the Beta coefficients (36). Non-healthcare workers were used as a reference group. The first analysis included a crude GEE model (model 1), followed by three models in which the covariates were added stepwise; model 2 (model 1 + sex, age, education level, household composition), model 3 (model 2 + employment contract, working hours), model 4 (model 3 + chronic health condition(s), chronic psychological illness, COVID-19 test result, COVID-19 vaccination). The covariate COVID-19 test result was included in all models as a time-varying variable, the other covariates were included as time-invariant variables. All analyses were conducted using IBM SPSS Statistics (version 25). A value of p of <0.05 was considered statistically significant.

## Results

### Study population

The study population consisted of 12,085 participants, including 2,750 healthcare workers and 9,335 non-healthcare workers (Figure 1). The percentage of females was higher (91.7%) among healthcare workers compared to non-healthcare workers (47.6%) (Table 1). Moreover, healthcare workers had less often a low education level (4.4%), compared to non-healthcare workers (13.9%). The mean age of healthcare workers and non-healthcare workers was 50.9 and 51.8 years, respectively.

**Table 1:** Characteristics of the study population stratified for healthcare workers and non-healthcare workers during the COVID-19 pandemic (March 2020–November 2021, *n* = 12,085 participants).

	Healthcare workers (*n* = 2,750)		Non-healthcare workers (*n* = 9,335)	
	Mean or %	SD or n	Mean or %	SD or n
Sex (% female/*n*)^*^	91.7	2,523	47.6	4,447
Age (in years) (mean/SD)^*^	50.9	9.2	51.8	8.2
18–35 (%/*n*)	8.9	244	4.9	458
36–50 (%/*n*)	29.7	816	31.5	2,944
51–67 (%/*n*)	61.5	1,690	63.6	5,933
Education level (%/*n*)^*^				
Low	4.4	120	13.9	1,301
Middle	46.9	1,289	39.3	3,670
High	48.8	1,341	46.7	4,364
Household composition (%/*n*)				
Living alone	8	219	8.7	813
Living with child(ren)	1.3	36	1.4	131
Living with adult(s)	55.3	1,520	54.8	5,116
Living with child(ren) and adult(s)	34.6	951	33.9	3,164
Living together but unknown with whom	0.9	24	1.2	111
Employment contract during COVID-19 pandemic (%/*n*)^*^				
Permanent	81.5	2,242	73.8	6,886
Temporary	7.5	207	14.9	1,388
Both permanent and temporary	10.9	301	11.4	1,061
Working hours per week (mean/SD)^*^	26.1	7.8	32.4	9.9
Occupational class (%/n)^*^				
High-skilled white-collar	77.2	2,124	54.6	5,099
Low-skilled white-collar	22.8	626	29.5	2,754
High-skilled blue-collar	–	–	8.1	759
Low-skilled blue-collar	–	–	7.7	723
Chronic health condition(s) (% yes/*n*)^*^	30.4	836	26.9	2,512
Chronic psychological illness (% yes/*n*)^*^	2.2	61	1.6	145
COVID-19 test result^1^ (% positive/*n*)^*^	10.5	289	7.9	741
COVID-19 vaccination (% yes/*n*)^*^	88.3	2,429	79.1	7,386

^1^In at least one of the questionnaire rounds.

^*^ Statistically significant (*p* < 0.05) difference of the characteristic between healthcare workers and non-healthcare workers tested with independent-samples *t*-test and chi-square test.

SD, standard deviation.

The percentages of participants testing positive for COVID-19 was higher among healthcare workers compared to non-healthcare workers (10.5% vs. 7.9% respectively) and healthcare workers were more often vaccinated against SARS-CoV-2 (88.3% vs. 79.1% respectively). The majority of characteristics were significantly different (*p* < 0.05) between healthcare workers and non-healthcare workers (Table 1).

### Anxiety symptoms over time

**Figure 2:**
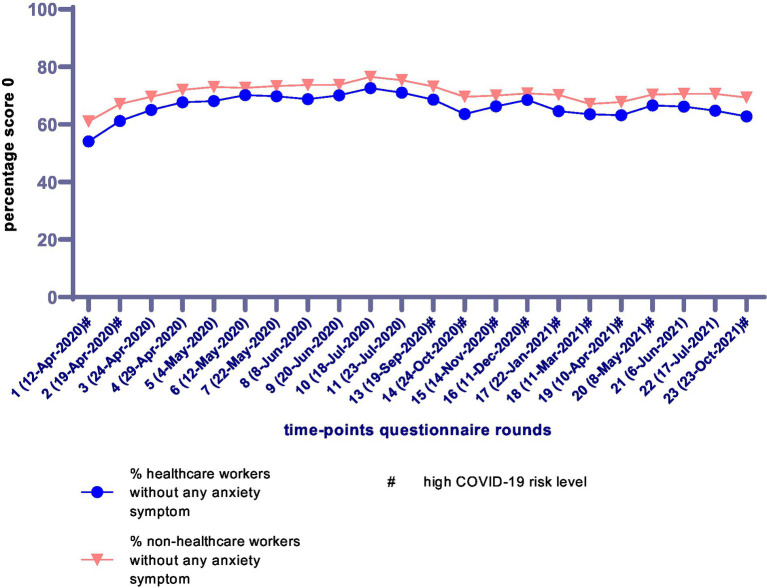
Percentages of participants without any anxiety symptoms over the different COVID-19 questionnaire rounds (March 2020–November 2021), stratified for healthcare workers (*n* = 2,750) and non-healthcare workers (*n* = 9,335). # High COVID-19 risk level (>100 COVID-19 associated hospitalizations per day) (37).

**Figure 3:**
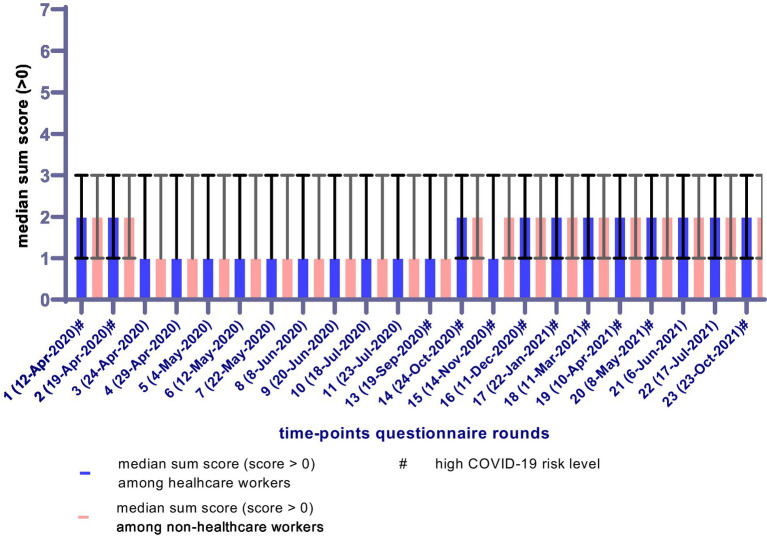
Median (and IQR) anxiety scores (for scores >0) over the different COVID-19 questionnaire rounds (March 2020–November 2021), stratified for healthcare workers (*n* = 2,750) and non-healthcare workers (*n* = 9,335). # High COVID-19 risk level (>100 COVID-19 associated hospitalizations per day) (37).

Figure 2 shows the anxiety sum scores over time by presenting the percentages of participants without any anxiety symptom (sum score = 0). The percentages of participants without any anxiety symptom were relatively low in the first questionnaire rounds and ranged from 54.1–72.6% over time for healthcare workers as compared to 61.0–76.5% for non-healthcare workers. Figure 3 shows the median anxiety sum score for scores >0. There was some variation in the median sum score of anxiety over time, but the scores for healthcare workers and non-healthcare workers were comparable. Across all questionnaire rounds, the median sum score of anxiety varied between 1 and 2 (with IQR = 1–3 in every round). During the periods with a high COVID-19 risk level, the median anxiety score was most often 2 for both healthcare workers and non-healthcare workers.

### Anxiety in healthcare and non-healthcare workers

In the crude GEE model, the incidence rate ratio (IRR) for the anxiety score during the COVID-19 pandemic was different for healthcare and non-healthcare workers. Following this model, healthcare workers scored on average 1.15 times higher on the anxiety score compared to non-healthcare workers [IRR = 1.15, 95% confidence interval (CI) = 1.08–1.22] (model 1, Table 2). However after adjusting for demographic covariates, differences between healthcare workers and non-healthcare workers in the anxiety score during the COVID-19 pandemic were no longer observed (model 2, Table 2). The full model (model 4, Table 2) including demographic, work and health characteristics was the best fitting model and also shows that the incidence rate ratio did not differ between healthcare workers and non-healthcare workers [incidence rate ratio (IRR) = 0.97, 95% (CI) = 0.91–1.04].

**Table 2:** Incidence rate ratios from the negative binomial GEE analyses on the longitudinal association between working in healthcare and the anxiety score during the COVID-19 pandemic, based on 22 measurement rounds (March 2020–November 2021, *n* = 12,085 participants).

	IRR	95% Confidence interval	value of *p*
Model 1: crude model	1.15	1.08–1.22	<0.001
Model 2: model 1 + demographic characteristics	0.99	0.93–1.06	0.811
Model 3: model 2 + work characteristics	0.98	0.92–1.04	0.474
Model 4: model 3 + health characteristics	0.97	0.91–1.04	0.383

Non-healthcare workers is the reference group. Demographic characteristics are sex, age, education level and household composition. Work characteristics are employment contract and working hours. Health characteristics are chronic health condition(s), chronic psychological illness, COVID-19 test result and COVID-19 vaccination.

## Discussion

The aim of the current study was to investigate the longitudinal association between working in healthcare and anxiety symptoms during the COVID-19 pandemic. We observed no differences in anxiety symptoms between healthcare workers and non-healthcare workers during the COVID-19 pandemic. The anxiety scores over time during the COVID-19 pandemic were similar for healthcare workers and non-healthcare workers.

The results did not confirm our hypothesis that healthcare workers experienced anxiety to a greater extent than non-healthcare workers during the COVID-19 pandemic, which was consistent with results from Chinese and Iranian studies (3, 38). These cross-sectional studies found no differences between healthcare workers and non-healthcare workers in their likelihood of experiencing anxiety during the COVID-19 pandemic as well. In the current study, the median anxiety sum score for both healthcare workers and non-healthcare workers was in general somewhat higher during peak periods of COVID-19 associated hospitalizations. This is in line with previous studies on mental health complaints during different periods of the COVID-19 pandemic (39–41). Yet, as periods with a high COVID-19 risk level occurred mostly in autumn and winter, seasonal variation could also have contributed to the higher anxiety score. To explain, research has shown that during autumn and winter, mental health complaints may occur more frequently among the general population (42–44).

Further, the results showed that before the demographic characteristics sex, age, education level and household composition were added to the model, healthcare workers scored on average 1.15 times higher on the anxiety score compared to non-healthcare workers. This could be due to the fact that being female is a major predictor of higher anxiety during the COVID-19 pandemic (45, 46). 91.7% of the healthcare workers in our sample was female and this percentage was considerably lower among the non-healthcare workers in our sample (47.6%).

A potential explanation for the fact that we found no differences in anxiety between healthcare workers and non-healthcare workers, could be that the working population in general was faced with uncertainty and risk of infection with SARS-CoV-2 (47, 48). A study from MacDonald et al. (49) found that intolerance of uncertainty and worries about contracting SARS-CoV-2 were associated with anxiety and other mental health problems among American adults.

Another possible explanation is that healthcare workers may have felt better informed about the pandemic than non-healthcare workers, which may have reduced negative mental health consequences of the pandemic among healthcare workers. Skoda et al. (22) found that the subjective level of being informed about COVID-19 and related measures was negatively associated with anxiety and that healthcare workers had a higher subjective level of information regarding COVID-19 than non-healthcare workers. In addition, a study from Cai et al. (50) showed that knowledge about COVID-19 and how to prevent the viral infection reduced distress among healthcare workers. If healthcare workers felt better informed and had more knowledge about COVID-19 compared to non-healthcare workers, this may have canceled out the negative effects of the COVID-19 pandemic on anxiety for healthcare workers.

In addition, the negative impact of the pandemic on healthcare workers might have been reduced by adequate preventive mental health care which was offered to healthcare workers during the COVID-19 pandemic (51). For example, activities such as mindfulness and psychosocial counseling at work and a specially opened national helpline to speak to specialized psychologists were offered to healthcare workers.

This study was one of the first longitudinal studies on anxiety among healthcare workers and non-healthcare workers during the COVID-19 pandemic. Anxiety was measured in periods of high and low COVID-19 infection rates, providing a complete overview of the situation in the first one-and-a-half year (March 2020–November 2021) of the pandemic in the Netherlands. Additional strengths are the large sample size, and the broad range of covariates that were included.

The study also has some limitations. First, the study population only consists of residents of the northern part of the Netherlands. This region had relatively low COVID-19 infection and mortality rates compared to other regions (52, 53). Because more severely affected regions were not examined, the degree of anxiety during the pandemic in the Netherlands may thus have been underestimated in the current study. Second, we were not able to distinguish between type of healthcare workers, while it is likely that healthcare workers in COVID-19 specific intensive care units experienced more anxiety, for example through being in contact with sick or deceased patients, than healthcare workers who were not in direct contact with COVID-19 patients. Third, nonresponse bias possibly occurred if workers (including those in healthcare) experiencing high levels of anxiety or mental health problems have not completed the surveys. Fourth, we used single dataset imputation for the 1 item missingness in rounds 1–9, whereas multiple imputation would have been more accurate.

Further longitudinal research could be insightful as some mental health problems may develop after a longer period of time (54–56). The SARS outbreak in 2003 showed that anxiety and other mental health problems in healthcare workers can persist and even increase long after the event (57). McAlonan et al. (57) explained these post-event complaints by the ending of direct threat and the allowance of suppressed emotions. Therefore, it is important to pay attention to the impact of the COVID-19 pandemic on the mental health of healthcare workers, for example by offering psychological help or support from the occupational physician, also after the pandemic.

## Conclusion

During the COVID-19 pandemic, many healthcare workers faced extreme working conditions and were at higher risk of being infected with SARS-CoV-2. Notwithstanding, we found no differences between healthcare workers and non-healthcare workers in their likelihood of experiencing anxiety symptoms during the COVID-19 pandemic. Considering the current and future high workload and workforce shortages especially in the healthcare sector (58, 59), it is important to continue monitoring the mental health of healthcare workers for the long term.

## Data availability statement

The data analyzed in this study is subject to the following licenses/restrictions: obtained from a third party, i.e., Lifelines (https://www.lifelines.nl/researcher). Requests to access these datasets should be directed to Lifelines Research Office (research@lifelines.nl).

## Ethics statement

The studies involving humans were approved by the UMCG Medical ethical committee. The studies were conducted in accordance with the local legislation and institutional requirements. The participants provided their written informed consent to participate in this study.

## Author contributions

VF and EB performed the data analyses. VF wrote the first draft of the manuscript. EB wrote the final draft of the manuscript. All authors contributed to the study conception, design, commented on previous versions of the manuscript, read, and approved the final manuscript.

## Group members of Lifelines Corona Research Initiative

H. Marike Boezen^1†^, Jochen O. Mierau^2,3,9^, H. Lude Franke^4^, Jackie Dekens^4,6^, Patrick Deelen^4^, Pauline Lanting^4^, Judith M. Vonk^1^, Ilja Nolte^1^, Anil P.S. Ori^4,5^, Annique Claringbould^4^, Floranne Boulogne^4^, Marjolein X.L. Dijkema^4^, Henry H. Wiersma^4^, Robert Warmerdam^4^, Soesma A. Jankipersadsing^4^, Irene van Blokland^4,7^, Geertruida H. de Bock^1^, Judith GM Rosmalen^5,8^, Cisca Wijmenga^4^

^1^Department of Epidemiology, University of Groningen, University Medical Center Groningen, Groningen, The Netherlands

^2^Department of Economics, Econometrics & Finance, Faculty of Economics and Business, University of Groningen, Groningen, The Netherlands

^3^Lifelines Cohort Study and Biobank, Groningen, The Netherlands

^4^Department of Genetics, University of Groningen, University Medical Center Groningen, Groningen, The Netherlands

^5^Department of Psychiatry, University of Groningen, University Medical Center Groningen, Groningen, The Netherlands

^6^Center of Development and Innovation, University of Groningen, University Medical Center Groningen, Groningen, The Netherlands

^7^Department of Cardiology, University of Groningen, University Medical Center Groningen, Groningen, The Netherlands

^8^Department of Internal Medicine, University of Groningen, University Medical Center Groningen, Groningen, The Netherlands

^9^Team Strategy & External Relations, University of Groningen, University Medical Center Groningen, The Netherlands

^†^Deceased
