# Supplementary material for: Anxiety among healthcare workers during the COVID-19 pandemic: a longitudinal study
Source: Front Public Health. 2023 Nov 30;11:1236931. doi: 10.3389/fpubh.2023.1236931 (PMC10720981; doi:10.3389/fpubh.2023.1236931)
Supplement: Supplementary file 1 [file Table_1.docx]

| *Table 3. Dates and number of respondents in the Lifelines COVID-19 questionnaire rounds.* | | | |
| --- | --- | --- | --- |
| Questionnaire round | Date | Number of respondents (n) | Number of included respondents (n) |
| 1 | 30-03-2020 to 23-04-2020 | 53,159 | 10,791 |
| 2 | 02-04-2020 to 06-05-2020 | 50,648 | 11,036 |
| 3 | 12-04-2020 to 06-05-2020 | 49,500 | 10,869 |
| 4 | 16-04-2020 to 13-05-2020 | 47,210 | 10,931 |
| 5 | 19-04-2020 to 20-05-2020 | 45,384 | 10,927 |
| 6 | 28-04-2020 to 27-05-2020 | 42,830 | 10,744 |
| 7 | 15-05-2020 to 29-05-2020 | 44,622 | 10,983 |
| 8 | 23-05-2020 to 24-06-2020 | 38,207 | 12,085 |
| 9 | 11-06-2020 to 29-06-2020 | 36,597 | 10,172 |
| 10 | 07-07-2020 to 29-07-2020 | 32,959 | 9,307 |
| 11 | 10-07-2020 to 05-08-2020 | 35,077 | 9,597 |
| 13 | 08-09-2020 to 30-09-2020 | 35,267 | 9,898 |
| 14 | 13-10-2020 to 04-11-2020 | 33,853 | 9,655 |
| 15 | 02-11-2020 to 26-11-2020 | 33,835 | 9,645 |
| 16 | 02-12-2020 to 21-12-2020 | 31,452 | 9,179 |
| 17 | 05-01-2021 to 08-02-2021 | 33,620 | 9,685 |
| 18 | 25-02-2021 to 25-03-2021 | 31,950 | 9,672 |
| 19 | 29-03-2021 to 22-04-2021 | 29,187 | 8,985 |
| 20 | 26-04-2021 to 20-05-2021 | 29,033 | 8,925 |
| 21 | 25-05-2021 to 18-06-2021 | 29,309 | 8,898 |
| 22 | 05-07-2021 to 29-07-2021 | 24,047 | 7,429 |
| 23 | 11-10-2021 to 04-11-2021 | 23,063 | 7,060 |
