# Supplementary material for: Anxiety among healthcare workers during the COVID-19 pandemic: a longitudinal study
Source: Front Public Health. 2023 Nov 30;11:1236931. doi: 10.3389/fpubh.2023.1236931 (PMC10720981; doi:10.3389/fpubh.2023.1236931)
Supplement: Supplementary file 2 [file Table_2.docx]

| *Table 4. A distribution of the coding across occupational classes according to the Occupational Classification of Statistic Netherlands (BRC ROA-CBS, 2014).* | |
| --- | --- |
| **Code** | **Occupational class** |
| 1 | Pedagogical occupations |
| 2 | Creative and linguistic occupations |
| 3 | Commercial occupations |
| 4 | Business economics and administrative occupations |
| 5 | Managers |
| 6 | Public administration, security and legal occupations |
| 7 | Technical occupations |
| 8 | ICT occupations |
| 9 | Agricultural occupations |
| 10 | Care and welfare occupations |
| 11 | Service occupations |
| 12 | Transport and logistics occupation |
| 13 | Not elsewhere classified |
