# Supplementary material for: Anxiety among healthcare workers during the COVID-19 pandemic: a longitudinal study
Source: Front Public Health. 2023 Nov 30;11:1236931. doi: 10.3389/fpubh.2023.1236931 (PMC10720981; doi:10.3389/fpubh.2023.1236931)
Supplement: Supplementary file 3 [file Table_3.docx]

| *Table 5. Classification of occupational sectors according to the Standard Business Classification (SBI), a classification of economic activities designed by Statistics Netherlands (2021).* | |
| --- | --- |
| **Code** | **Sector** |
| 1 | Agriculture |
| 2 | Mineral extraction |
| 3 | Industry |
| 4 | Energy |
| 5 | Water |
| 6 | Construction |
| 7 | Commerce |
| 8 | Transport and storage |
| 9 | Hotels and catering |
| 10 | Information and communication |
| 11 | Finance |
| 12 | Real estate |
| 13 | Business services |
| 14 | Public services |
| 15 | Education |
| 16 | Health services |
| 17 | Culture, sports and recreation |
| 18 | Other |
