# Supplementary material for: Anxiety among healthcare workers during the COVID-19 pandemic: a longitudinal study
Source: Front Public Health. 2023 Nov 30;11:1236931. doi: 10.3389/fpubh.2023.1236931 (PMC10720981; doi:10.3389/fpubh.2023.1236931)
Supplement: Supplementary file 4 [file Table_4.docx]

| *Table 6. The Mini-International Neuropsychiatric Interview items and the corresponding questions in the Lifelines data.* | | |
| --- | --- | --- |
| **Item** | **Mini-International Neuropsychiatric Interview** | **Question in the Lifelines data** |
| 1 | Have you worried excessively or been anxious about several things over the past 6 months? | In the last 7/14 days, have you been worrying excessively and worrying about multiple problems of everyday life, at work, at home, in your immediate environment? |
| 1a | Are these worries present most days? | Were these worries present almost every day in the last 7/14 days? |
| 1b | Do you find it difficult to control the worries or do they interfere with your ability to focus on what you are doing? | Do you find it difficult to control the worries or do they interfere with your ability to focus on what you are doing? |
| 2 | When you were anxious over the past 6 months, did you, most of the time feel restless, keyed up or on edge? | In the last 7/14 days did it often happen that you felt restless, jittery or nervous? |
| 3 | When you were anxious over the past 6 months, did you, most of the time feel tense? | In the last 7/14 days did it often happen that you felt tense? |
| 4 | When you were anxious over the past 6 months, did you, most of the time feel tired, weak or exhausted easily? | In the last 7/14 days did you feel tired or without energy almost every day? |
| 5 | When you were anxious over the past 6 months, did you, most of the time have difficulty concentrating or find your mind going blank? | In the last 7/14 days was it difficult to concentrate or make decisions almost every day? |
| 6 | When you were anxious over the past 6 months, did you, most of the time feel irritable? | In the last 7/14 days did it often happen that you were particularly irritable? |
| 7 | When you were anxious over the past 6 months, did you, most of the time have difficulty sleeping (difficulty falling asleep, waking up in the middle of the night, early morning wakening or sleeping excessively)? | In the last 7/14 days have you had problems sleeping almost every night (difficulty falling asleep, waking up in the night or too early in the morning, or actually sleeping too much)? |
| Note: Items 1a and 1b were dependent questions on item 1. Therefore, items 1a and 1b were not included in the anxiety sum score. | | |
